# Optimizing multi-domain hematologic biomarkers and clinical features for the differential diagnosis of unipolar depression and bipolar depression

**DOI:** 10.1038/s44184-023-00024-z

**Published:** 2023-04-10

**Authors:** Jinkun Zeng, Yaoyun Zhang, Yutao Xiang, Sugai Liang, Chuang Xue, Junhang Zhang, Ya Ran, Minne Cao, Fei Huang, Songfang Huang, Wei Deng, Tao Li

**Affiliations:** 1 Hangzhou Seventh People’s Hospital, Affiliated Mental Health Center, School of Medicine, Zhejiang University, Hangzhou, Zhejiang China; 2 Alibaba Damo Academy, 969 West Wen Yi Road, Yu Hang District, Hangzhou, Zhejiang China; 3 Center for Cognition and Brain Sciences, Unit of Psychiatry, Institute of Translational Medicine, University of Macau, Macao, China; 4 West China Hospital, Sichuan University, Sichuan, China; 5 Department of Neurobiology, Affiliated Mental Health Center & Hangzhou Seventh People’s Hospital, Zhejiang University School of Medicine, Hangzhou, China; 6 Liangzhu Laboratory, MOE Frontier Science Center for Brain Science and Brain-machine Integration, State Key Laboratory of Brain-machine Intelligence, Zhejiang University, 1369 West Wenyi Road, 311121 Hangzhou, China; 7 NHC and CAMS Key Laboratory of Medical Neurobiology, Zhejiang University, 310058 Hangzhou, China

**Keywords:** Diagnostic markers, Bipolar disorder, Depression

## Abstract

There is a lack of objective features for the differential diagnosis of unipolar and bipolar depression, especially those that are readily available in practical settings. We investigated whether clinical features of disease course, biomarkers from complete blood count, and blood biochemical markers could accurately classify unipolar and bipolar depression using machine learning methods. This retrospective study included 1160 eligible patients (918 with unipolar depression and 242 with bipolar depression). Patient data were randomly split into training (85%) and open test (15%) sets 1000 times, and the average performance was reported. XGBoost achieved the optimal open-test performance using selected biomarkers and clinical features—AUC 0.889, sensitivity 0.831, specificity 0.839, and accuracy 0.863. The importance of features for differential diagnosis was measured using SHapley Additive exPlanations (SHAP) values. The most informative features include (1) clinical features of disease duration and age of onset, (2) biochemical markers of albumin, low density lipoprotein (LDL), and potassium, and (3) complete blood count-derived biomarkers of white blood cell count (WBC), platelet-to-lymphocyte ratio (PLR), and monocytes (MONO). Overall, onset features and hematologic biomarkers appear to be reliable information that can be readily obtained in clinical settings to facilitate the differential diagnosis of unipolar and bipolar depression.

## Introduction

Mood is defined as a pervasive and persistent tone of feeling that is endured internally and impacts nearly all aspects of a person’s behavior in the external world. Mood disorders, also known as affective disorders, are described as marked disturbances in mood - severe lows called depression or highs called (hypo)mania^1^. Mood disorders are common mental illnesses that lead to increased morbidity and mortality^1^. In the International Classification of Diseases 10th edition (ICD-10) and the Diagnostic and Statistical Manual of Mental Disorders 4th edition (DSM-IV), mood disorders mainly include major depressive disorder, bipolar disorder, persistent mood disorder, and cyclothymic disorder^2,3^. However, in the new classification criteria of the fifth edition of the DSM (DSM-5-TR)^4^ and the synchronized ICD-11^5–7^, mood disorders are considered as two separate categories, depressive disorders and bipolar disorders. As part of the mixed categorical-dimensional approach, DSM-5-TR also includes multiple specifiers to describe depressive disorders and bipolar disorders in more detail^4^.

Major depressive depression, also known as unipolar depression (UPD), is a serious mental disorder. Its main clinical features are low mood, lack of interest, and loss of pleasure, accompanied by loss of appetite, sleep disorders, low self-evaluation, and pessimistic world-weariness. There are also changes in patients’ cognition and behavior^8^. In contrast, bipolar depression (BPD) refers to a depressive episode of bipolar disorder (BD)^9^. Bipolar disorder (BD) is characterized by alternating episodes of depression and (hypo)mania^4^. In particular, BD I and BD II are two main categories of BD^4,9^. They differ essentially in whether the patient experiences manic episodes (BD I) or only hypomanic episodes (BD II)^4^. Patients with UPD and BPD have many similar clinical manifestations, and depressed mood is the most important manifestation of both^4^. Notably, most patients diagnosed with BD spend more time in the depressive episode than in the (hypo)manic episode^9,10^. In addition, onset of BD often begins with a depressive episode, and patients with BD may not have a clear history of (hypo)manic episodes early in the course^9–11^. Besides, there is a lack of epidemiological features or subliminal symptoms of (hypo)manic that can assist in differential diagnosis^12–15^. Therefore, (hypo)manic episodes are difficult to catch and are frequently overlooked by doctors and patients, especially in BD II, where hypomanic episodes are relatively mild and more difficult to detect^7,12–14,16^. Other clinical symptom features used to differentiate the two diseases are usually of low performance (in terms of accuracy, sensitivity, and specificity) and are not sufficient for clinical practice^17,18^. Therefore, it is challenging to distinguish between unipolar and bipolar depression, especially during the onset of the illness^19,20^. Surveys have shown that only 20% of BPD patients can be correctly diagnosed at the onset, and it takes an average of 10-15 years for patients to be correctly diagnosed^21–23^. On the other hand, despite the clinical manifestations of UPD and BPD are very similar, the clinical outcomes and treatment options are completely different. For UPD, antidepressants are used clinically to treat depressive symptoms and prevent their relapse. In the case of BPD, in addition to treating depressive symptoms, prevention of (hypo)manic episodes is also required and thus it is mainly treated with mood stabilizers and/or atypical antipsychotics^24,25^. BPD is often misdiagnosed as UPD, leading to incorrect treatment with unopposed antidepressants. Antidepressants are often ineffective in treating BPD and may lead to deleterious outcomes such as (hypo)manic during treatment, rapid cycling, or increased suicide rates^26^.

Considering that most patients with onset of BD have depressive episodes and that depressive episodes remain predominant throughout the disease course (the number of depressive episodes is about 3 times the number of (hypo)manic episodes)^27,28^, it is necessary to find objective and easily accessible features/biomarkers to differentiate between UPD and BPD, and establish high-performance diagnostic models with strong discriminative power in a broad patient coverage that can be widely disseminated in clinical settings.

Considering the clinical features of the differential diagnosis of UPD and BPD, the psychiatric symptoms used in the DSM-5-TR diagnostic guidelines are subjective and especially ambiguous in the early stages^10,29^. Correspondingly, scales for mental health ratings suffer from time-consuming, subjective answers, and inconsistencies among raters, which are not routinely used in clinicians’ daily practice^30^. On the other hand, world-wide epidemiological studies have found statistical differences in the course characteristics of UPB and BPD. Compared with UPD, the onset age of BPD is earlier and the duration of disease is longer^29,31^. Age of onset and disease duration are objective, readily available clinical features with broad patient coverage that may have great potential for use as cost-effective differential diagnosis tools.

Besides, related works proposed to use biomarkers from different domains, such as serum levels^32^, MRI (Magnetic Resonance Imaging)^33–35^ and cognitive function^14^, for statistical and machine learning methods in the differential diagnosis of UPD and BPD. In particular, there are extensive studies and meta-analysis regarding longitudinal associations between inflammatory biomarkers such as CRP (C-reactive protein)/IL-6 (interleukin-6) and UPD^36–38^, and between blood-based protein biomarkers and BPD^39^. However, the problem with current approaches is that it is difficult to obtain such biomarkers in routine clinical practice. On the other hand, biomarkers of complete blood count (CBC) and blood biochemical markers (BCMs) can be conveniently obtained in clinics, through low-cost and reproducible tests that can be easily conducted under simple laboratory conditions.

Previous works have attempted to find associations between CBC biomarkers and mood disorders. For example, white blood cell count (WBC) is a nonspecific marker of inflammatory that is often measured as part of a CBC panel. The association between leukocyte subtypes and affective disorders has been demonstrated in previous studies^40,41^. In addition, neutrophil-to-lymphocyte ratio (NLR), platelet-to-lymphocyte ratio (PLR), and monocyteto-lymphocyte ratio (MLR) have recently been proposed as inflammatory markers. These biomarkers appear to be associated with mood disorders, supporting the inflammatory hypothesis underlying the etiopathogenesis of these conditions^40,42^. To date, several studies have examined the validity of NLR, PLR, and MLR as potential biomarkers for differentiating BPD from UPD^40,41^.

In terms of biochemical indicators, the body’s antioxidant defense system includes two categories: enzymatic and non-enzymatic antioxidants. Although the detection of enzymatic antioxidant substances such as superoxide dismutase and catalase are relatively difficult, the detection of non-enzymatic antioxidant substances such as albumin is included in the routine liver and kidney function tests and can be carried out conveniently. Albumin and other nonenzymatic antioxidants can be used to monitor the body’s antioxidant levels^43^. Previous studies have shown that the concentrations of plasma albumin and other non-enzymatic antioxidants are lower in patients with depression; concentration of albumin in the major depressive group was lower than that of the manic group, and both were lower than that of the control group^44^. Furthermore, an association between serum uric acid and depressive symptoms was identified^45,46^, and changes in blood lipid levels have also been reported to be associated with schizophrenia, UPD and bipolar manic^45,46^.

Despite unipolar and bipolar depression have different associations with clinical features such as the age of onset and hematologic biomarkers, none of previous works have used such information for differential diagnosis. It is urgent to establish objective features that can be easily accessible in practical settings and develop accurate differential diagnosis models for UPD and BPD^19,47^. Besides, combining differential traits/biomarkers from multiple domains is encouraged to better represent population heterogeneity originating from different aspects^48,49^. In this study, using clinical features and hematologic biomarkers, we took the initiative to build and validate an automated differential diagnostic model on a large scale cohort of 1160 eligible patients (918 UPD and 242 BPD) in practical settings.

## Methods

### Participants

This was a naturalistic, retrospective, cross-sectional study. All the patients were from Hangzhou Seventh People’s Hospital and completed blood-related examinations. Data from 2018.01–2021.06 was collected. All data available in that period were analyzed. Only the baseline CBC and biochemistry tests of the first entry for each patient from inpatient care units was used for analysis. Usually, the first blood tests are done next day after admission to our units when the patients were after 12 h of fasting. Thus, we have assumed that most patients that we included in this study were in acute phase of their disorder. We mainly included patients between the ages of 18 and 65 years who were diagnosed with BPD and UPD based on the ICD-10 criteria. Patients were grouped under diagnostic criteria as unipolar depression (F32 and F33 according to ICD-10) and bipolar depression (F31.3-F31.5 according to ICD-10).

Patients with diagnostic codes for other comorbid psychiatric disorders, such as anxiety, were excluded from the cohort. Besides, the distributions of potential confounders of smoking/alcohol status were examined and found to be independent from the two disorders and specific biomarkers based on our correlation analysis. Moreover, as a general rule, patients with coexisting severe somatic diseases (e.g., acute autoimmune and inflammatory diseases, renal failure, cancer, or other), which may significantly affect various blood parameters, were not included in our study. Thus, we assume that observed results are mostly related to psychiatric conditions.

**Fig. 1:**
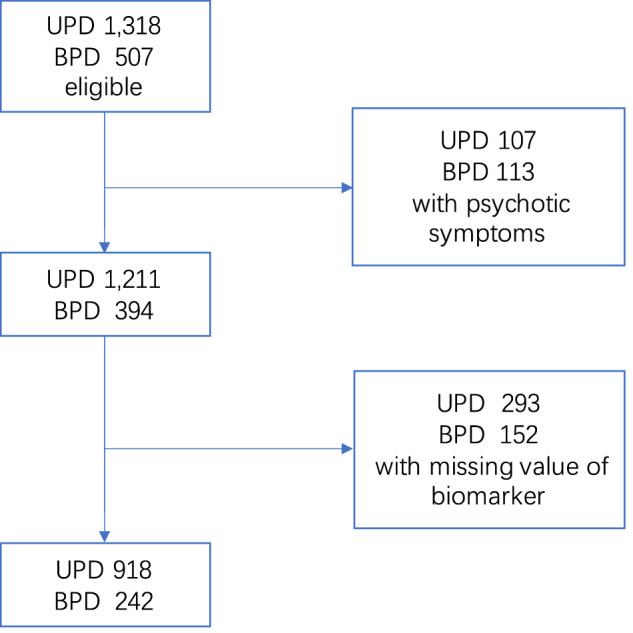
Flow of participants in the study. Patients with psychotic symptoms and missing values of biomarkers were excluded from the participants. As a result, a total of 921 UPD patients and 239 BPD patients participated in the study.

The initial number of patients with UPD is 1318, the initial number of patients with BPD is 507. As illustrated in Fig. 1, 107 patients of UPD with psychotic symptoms, and 113 patients of BPD with psychotic symptoms were removed from the cohort; 290 patients of UPD and 155 patients of BPD with missing values of any biomarkers were removed from the cohort. Finally, 921 and 239 patients left as participants in the study, respectively.

### Clinical and biological data acquisition

#### Clinical data acquisition

Basic socio-demographic and clinical features were obtained through electronic medical record system including age, gender, diagnosis, age of onset, and duration of the disease.

#### Blood data acquisition

Blood samples for CBC tests and blood biochemistry tests were taken in the morning (between 7 and 9 a.m.) of the first day of hospitalization, after 12 h of fasting, from a forearm vein. For each patient, about 3 ml of blood was collected in hemogram tubes containing EDTA. After collecting blood samples, CBC was determined using Sysmex XN-3000 Automated Hematology Analyzer (Sysmex, USA). Blood biochemistry was analyzed using Siemens automatic biochemical analyzer-XTP.

### Statistical analysis

Statistical analyses were conducted by IBM SPSS Statistics 22 (IBM SPSS, Turkey). The comparison of the sex distribution between the two groups was performed using χ2 test. Comparisons including age, age of onset, and total disease duration between the two groups were performed using a two-tailed two-sample t test (for examined normal distribution of data). Unless specified otherwise, the significance of all tests was set to *p* < 0.05.

### Machine learning process

**Fig. 2:**
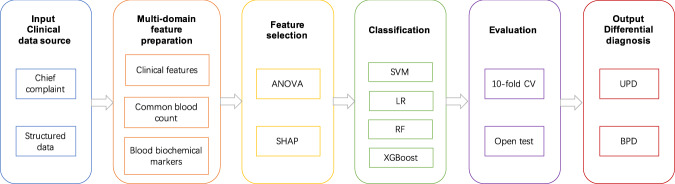
Study design of classification-based differential diagnosis between unipolar and bipolar depression. Features of three domains were first extracted from clinical data sources of chief complaints and structured tables. Then, two feature selection algorithms, ANOVA and SHAP, were adopted to select the most informative features. After that, four classification algorithms were used for differential diagnosis including support vector machine (SVM), logistic regression (LR), random forest (RF), and Extreme Gradient Boosting (XGBoost) methods. Models were trained using 10-fold cross-validation and further evaluated on the open-test dataset.

Figure 2 illustrates our study design for classification-based differential diagnosis of unipolar and bipolar depression: given clinical data sources of both unstructured text in the chief complaint and structured tables, features in three domains were extracted from all subjects, including clinical features, CBC biomarkers, and BCMs. Then, two feature selection algorithms, analysis of variance (ANOVA)^50^ and SHapley Additive exPlanations (SHAP)^51^, were adopted to select most informative features. Afterwards, four classification algorithms were used for differential diagnosis, including support vector machine (SVM), logistic regression (LR), random forest (RF), and Extreme Gradient Boosting (XGBoost) methods. Models were trained using 10-fold cross-validation (CV) and further evaluated on an open-test dataset. The final output is a diagnosis of unipolar or bipolar depression.

#### Feature preparation

**Fig. 3:**
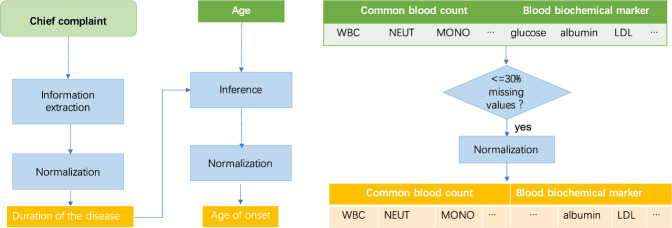
Workflow of feature preparation. Two clinical features, duration of the disease and age of the disease onset, were extracted and calculated based on chief complaints; common blood biomarkers and blood biochemical markers with more than 30% missing values were removed, remaining 37 biomarkers normalized into z-scores.

After a thorough survey of potential features for differential diagnosis between UPD and BPD identified in previous research and a discussion with physicians about the important information available in the hospital EHR system, features in three domains were prepared and examined in this study (Fig. 3):

1. two clinical features including duration of the disease and age of the disease onset. The chief complaints in clinical notes usually started with the major symptoms of patients and the total durations. Therefore, duration of the disease was first extracted from the free text of chief complaints using regular expressions of temporal patterns. For example, from the text of “*Sleep disturbances for 3 years 2 months*”., “*3 years 2 months*” was extracted and considered as the duration of the disease. Next, the age of the disease onset could be inferenced by subtracting the duration from the age of the patient. The disease duration was normalized in the unit of year, and the age of onset was normalized into four groups—1 for age ≤20, 2 for age ≤40 and >20, 3 for age ≤60 and >40, and 4 for age ≤65 and >60.
2. 27 CBC biomarkers including (a) biomarkers of the leukocyte system (WBC markers): WBC, MONO, MONO ratio, NEUT, NEUT ratio, BASO, BASO ratio, EO, EO ratio, LYMPH, LYMPH ratio; (b) biomarkers of the erythrocyte system (RBC markers): RBC, HCT, MCV, RDW-CV, HGB, MCH, MCHC, RDW-SD; (c) biomarkers of the platelet system (platelet markers): PLT, PDW, MPV, P-LCR; (d) Blood count-related inflammatory markers: NLR, PLR, MLR, which can be used to evaluate the inflammatory state of the body.
3. 17 BCMs including a) electrolyte markers: calcium, chloride, potassium, magnesium, sodium, phosphorus; b) protein markers: globulin ratio, albumin, total protein, globulin; (c) markers of kidney function including creatinine, urea nitrogen; (d) marker of blood sugar - glucose; e) markers of blood lipids: LDL, HDL, triglycerides, and total cholesterol.

Abbreviations of blood biomarkers used in this study and their full names are listed in Supplementary Table 1. Biomarkers with more than 30% missing values were removed (magnesium, globulin ratio, total protein, nitrogen, glucose, triglycerides, total cholesterol). The remaining 37 biomarkers were normalized into z-scores and went through feature selection in this study.

#### Feature selection

In order to select effective features and improve the disease classification performance, two feature selection algorithms, ANOVA^50^ and SHAP^51^, were used on each classifier. ANOVA measures the relevance of features to the categories (i.e., UPD and BPD) by determining whether their means come from the same distribution or not, whereas a SHAP value for a feature of a specific prediction represents how much the model prediction changes when we observe that feature.

#### Machine learning methods

1. LR: LR estimates the parameters of a logistic model; it is a form of binomial regression.
2. SVM: SVM is based on the statistical learning theory of the VC dimension and the structural risk minimization principle. SVMs use kernel functions (such as radial basis kernel functions and linear kernel functions) to project high-dimensional samples into lower dimensions to improve the prediction or classification ability of the model.
3. RM: RM is an ensemble learning method that operates by constructing multiple decision trees during training.
4. XGBoost: XGBoost is a scalable tree-based gradient boosting algorithm. It generates accurate predictions by integrating weak classifiers.

### Evaluation

#### Gold standard

In this study, ICD10^2^ was used as the diagnostic standard for mental disorders, and discharge diagnosis was used as the gold standard for diagnosis. Specifically, discharge diagnoses were obtained from follow-up visits by senior physician during ward rounds. At least two senior physicians should follow up and agree on the diagnosis. If diagnostic discrepancies could not be resolved, difficult cases were discussed in groups until a consistent diagnosis was made.

Inter-rater agreement between the gold standard and the structured clinical diagnosis of CIDI (Comprehensive International Diagnostic Interview)^52^ was calculated using 100 samples randomly selected from the cohort. Cohen’s kappa value was reported to be 0.83, indicating that the gold standard is fully consistent with the structured clinical diagnosis of CIDI.

#### Evaluation criteria

Standard metrics, i.e., the area under the receiver operating characteristic curve (AUC), sensitivity, specificity and accuracy, were reported to evaluate the classification performance of different models. The AUC usually provides a view of performance stability. In a general situation, an AUC of 0.90–1.0 is regarded as very high (excellent), of 0.80–0.89 high (good), of 0.70–0.79 moderate (fair), of 0.60–0.69 low (poor), and of 0.50–0.59 as very low (fail or useless)^39^. Meanwhile, sensitivity, specificity and accuracy can provide a more objective model assessment from other aspects. Balanced sensitivity and specificity scores were reported based on the ROC (receiver operating characteristic) curves.

#### Experimental setup

The dataset was split into a training set (85%) and an open test set (15%) for differential diagnosis evaluation. To avoid bias in the data distribution, the dataset was randomly split into training and open test sets 1000 times, and the average performance was reported in this study. The differential diagnosis classification model was constructed using the training set, and the parameters were continuously adjusted to optimize the model through 10-fold CV. The final performance of each machine learning algorithm was evaluated using the open test set.

In our experiments, machine learning algorithms were implemented using the scikit-learn version 0.24.2 packages. Values of biomarkers were normalized to z-scores using the StandardScaler in scikit-learn. The optimal parameters were selected using GridSearchCV.

To demonstrate the discriminative contribution of each feature type and their combinations, differential diagnostic performance using clinical features, CBC biomarkers, BCM markers and their combinations (Clinical+CBC, Clinical+CBC + BCM) is reported. The statistical significance of the difference in AUC between using the entire feature set (Clinical+CBC + BCM) and the other feature sets is also calculated.

Performance of using clinical features, blood biomarker features and their combinations was reported, respectively. Moreover, to understand the effectiveness of the differential diagnosis model on patients at early/later stages of the disease course, performance on the samples with disease durations ≤3 years and >3 years was also reported, respectively.

### Ethical aspects

The study was carried out in accordance with ethical principles for medical research involving humans (WMA, Declaration of Helsinki). All data were collected anonymously. This retrospective study protocol was approved by the Ethics Committee of the Hangzhou Seventh People’s Hospital, with a granted waiver of informed consent.

## Results

### Participants

In total, 1160 inpatients of unipolar and bipolar depression were enrolled in the present study, of which 918 were experiencing UPD and 242 were experiencing BPD. The mean (±SD) age of the total sample was 36.86 (±15.04). 771 (66.47%) were females, and 89.31% (*N* = 1136) were employed. As for the total duration of the disease, the mean (±SD) length was 5.18 (±7.80). Other socio-demographic and clinical characteristics are displayed in Table 1.

**Table 1:** Socio-demographic and clinical characteristics of samples in this study.

Characteristic	Unipolar depression *N* = 918	Bipolar depression *N* = 242	*p*
Age^a^^ (years)	43.08 ± 14.67	37.79 ± 14.56	<0.001
Female^a^*	629 (0.685)	142 (0.587)	0.004
Age of onset^a^^ (years)	39.81 ± 14.60	25.62 ± 10.84	<0.001
Disease duration^a^^(years)	3.34 ± 5.612	12.14 ± 10.55	<0.001

^^^Values shown as mean and standard deviation.

*Values shown as count and percent of distribution.

^a^Unipolar depression is significantly different from bipolar depression.

The comparison of sex distributions was performed using χ2 test. Comparisons of age, age of onset and total disease duration were performed using a two-tailed two-sample t test.

**Fig. 4:**
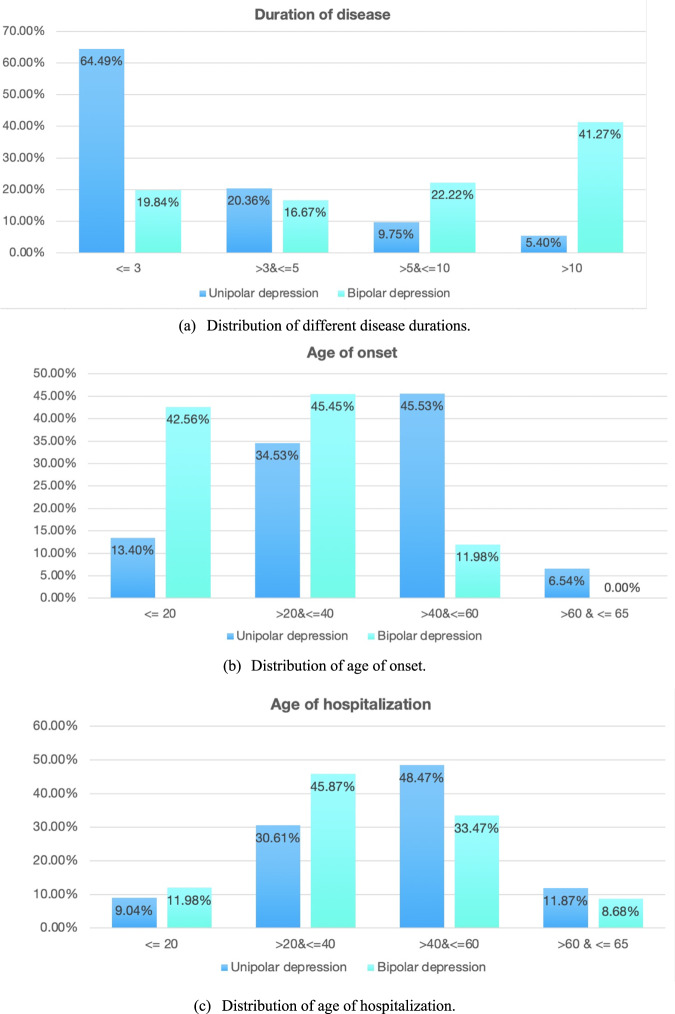
Sample distributions of unipolar and bipolar depression (Unit: year). **a** Distribution of different disease durations. **b** Distribution of age of onset. **c** Distribution of age of hospitalization.

Figure 4a illustrates the cohort size distributions of different durations of UPD and BPD. It is interesting to observe that a majority (~64%) of UPD patients had a duration ≤3 years. The percentages of patients with UPD (~20%) and BPD (~17%) were most close in the duration of 3–5 years, from where the percentage of BPD patients increased consistently up to ~41% in the duration of >10 years.

Figure 4b illustrates the distributions of ages of onset for UPD and BPD. ~43% of BPD patients had an early onset ≤20 years old and ~45% of them had an onset between 20 and 40. In contrast, the majority of onset ages of UPD were between 20 to 60. Taking a look at both Fig. 4a and b, we can find that BPD had a relatively earlier age of onset and a longer duration of disease, while UPD had a relatively later onset of the disease and a shorter duration. Such differences are typical for UPD and BPD, indicating that the cohort used in this study is representative.

Following Fig. 4a, b, Fig. 4c illustrates the distributions of ages of patient hospitalization for UPD and BPD. The majority patients were between 20 to 60 years old for both diseases (~79%). Notably, an approximate shape of symmetry could be observed for the age distributions of UPD and BPD patients. The hospitalized patients were relatively younger for BPD (~12% ≤20, ~46% between 20 and 40), while the hospitalized patients were relatively older for UPD (~48% between 40 and 60, ~12% between 60 and 65).

### Selected features for differential diagnosis

**Fig. 5:**
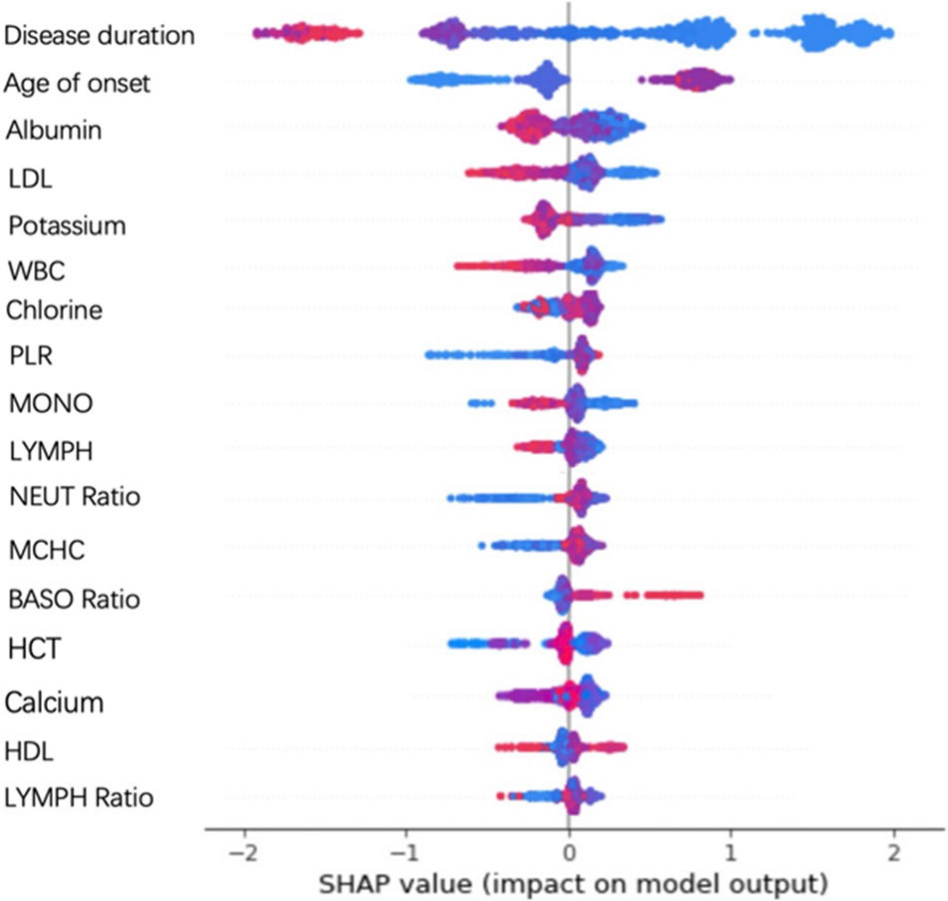
Dot plot of feature importance. Dot plot of feature importance calculated using the mean SHAP values from running 10-fold cross validation on the training set for 1000 times. The x-axis was the SHAP value (in unit of log odds) of the features used for diagnosis classification. Each row shows the importance of different values of one feature, with red color indicating high feature values and blue color indicating low feature values. The positive x-axis represents the importance of each feature value to support unipolar depression, and the negative axis represents the importance of each feature value to support bipolar depression. The rows were ranked by the overall feature importance vertically. Therefore, disease duration has the strongest drive to the model’s prediction, while LYMPH ratio has a relatively weaker drive. Notably, when points do not fit together on the line, they pile up vertically to show density.

Seventeen features top ranked by SHAP values were selected for classification in this study, which yielded the optimal performance with XGBoost. Both clinical features, disease duration and age of onset were considered as effective features. Selected CBC features included four WBC biomarkers—WBC, MONO, NEUT Ratio, and BASO Ratio, two RBC biomarkers—HCT and MCHC, two biomarkers of platelets—LYMPH and LYMPH Ratio, and one biomarker of inflammation - PLR. Six BCM markers were selected, including three electrolyte markers - potassium, chlorine, and calcium, one protein marker—albumin, and two markers of blood lipids - HDL and LDL. To further look into the importance of each feature, a summary plot was drawn with all the SHAP values for a single feature as depicted in Fig. 5. The same set of features were selected by ANOVA as the top seventeen features, with different orders of importance. A ranking of the entire feature set based on SHAP values can be found in Supplementary Fig. 1.

The x-axis in Fig. 5 was the SHAP value (in unit of log odds) of the features used for diagnosis classification. Each row shows the importance of different values of one feature, with red color indicating high feature values and blue color indicating low feature values. The rows were ranked by the overall feature importance vertically. Therefore, disease duration has the strongest drive to the model’s prediction, while LYMPH ratio has a relatively weaker drive. Notably, when points don’t fit together on the line, they pile up vertically to show density.

Blood biomarker levels may vary with clinical variables such as age, gender, age of onset and disease duration. As shown in Table 1, statistically significant differences in each of the four clinical variables are observed between the two groups. To examine the differential ability of blood biomarkers for UPD and BPD, especially while considering the potential influence of confounding clinical variables, blood biomarkers are compared between the two groups, taking the top 3 CBC biomarkers (WBC, PLR, MONO) and the top 3 BCM biomarkers (Albumin, LDL, Potassium) as examples. T-tests and tests of between-subjects effects are conducted with each blood biomarker as dependent variable and clinical variable as covariate. As illustrated in Table 2, statistically significant differences between the two groups are observed for each blood biomarker, regardless of whether clinical variables are considered as covariates. This demonstrates the independent ability of each biomarker to distinguish unipolar from bipolar depression. To examine the influence of covariant clinical features on differential diagnostic performance, we further added age and gender into the feature set. The performance of the resulting differential diagnosis models was compared with the original feature set. Please find a more detailed comparison in the Results section.

**Table 2:** Comparison of each blood biomarker of unipolar and bipolar depression between the two groups.

Biomarker	Unipolar depression	Bipolar depression	p_t	p_b
WBC	5.87 ± 1.59	6.67 ± 2.01	<0.001	<0.001
PLR	121.99 ± 46.76	111.24 ± 43.52	<0.001	<0.001
MONO	0.46 ± 0.15	0.52 ± 0.18	<0.001	<0.001
Albumin	40.77 ± 3.47	41.94 ± 4.11	<0.001	<0.001
LDL	2.47 ± 0.79	2.69 ± 0.88	<0.001	<0.001
Potassium	4.00 ± 0.33	4.07 ± 0.31	<0.001	<0.001

Two sets of p-values are reported: p_t is obtained from T-tests; p_b is obtained from Tests of between-subjects effects with each blood biomarker as dependent variable and clinical variables as covariates. Statistically significant differences are observed for each blood biomarker between the two groups, regardless of whether clinical variables are considered as covariates. This demonstrates the independent ability of each biomarker to differentiate between unipolar and bipolar depression.

### Performance of differential diagnosis

XGBoost using SHAP for feature selection achieved the optimal performance, which was reported in Tables 3–5. ROC curves of XGBoost were illustrated in Supp Fig. 2. Performance of other algorithms was reported in Supplementary Tables 2–4.

**Table 3:** Classification performance of XGBoost using clinical features, hematologic biomarkers and their combination, based on the entire cohort.

Feature	Dataset	Accuracy	Sensitivity	Specificity	AUC
**Clinical**	10fold_CV**	0.827	0.807	0.782	0.830
	Open test**	0.810	0.830	0.795	0.875
**CBC**	10fold_CV**	0.682	0.676	0.698	0.700
	Open test**	0.658	0.638	0.658	0.667
**BCM**	10fold_CV**	0.550	0.563	0.502	0.513
	Open test**	0.590	0.581	0.525	0.544
**Clinical** + **CBC**	10fold_CV**	0.836	0.812	0.828	0.844
	Open test**	0.840	0. 848	0.810	0.883
**Clinical** + **CBC** + **BCM**	10fold_CV	0.843	0.816	0.831	0.850
	Open test	0.850	0. 850	0.816	0.889

Comparisons of AUCs between the combined features and other feature groups were performed using T-tests based on 1000 permutations. A statistically significant difference is noted: *P-value < 0.05; **P-value < 0.001. *CBC* Complete blood count, *BCM* Blood biochemical marker.

**Table 4:** Classification performance of XGBoost using clinical features, hematologic biomarkers and their combination, based on samples of disease duration ≤ 3 years.

Feature	Dataset	Accuracy	Sensitivity	Specificity	AUC
**Clinical**	10fold_CV*	0.930	0.785	0.732	0.795
	Open test*	0.940	0.800	0.725	0.817
**CBC**	10fold_CV**	0.910	0.665	0.608	0.702
	Open test**	0.920	0.678	0.658	0.719
**BCM**	10fold_CV**	0.890	0.608	0.499	0.625
	Open test**	0.900	0.615	0.502	0.627
**Clinical** + **CBC**	10fold_CV**	0.930	0. 796	0.741	0.812
	Open test**	0.940	0. 815	0.765	0.847
**Clinical** + **CBC** + **BCM**	10fold_CV	0.940	0. 800	0.754	0.829
	Open test	0.950	0. 820	0.775	0.859

Comparisons of AUCs between the combined features and other feature groups were performed using T-tests based on 1000 permutations. A statistically significant difference is noted: *P-value < 0.05; **P-value < 0.001. *CBC* Complete blood count, *BCM* Blood biochemical marker.

**Table 5:** Classification performance of XGBoost using clinical features, hematologic biomarkers and their combination, for samples of disease duration >3 years.

Feature	Dataset	Accuracy	Sensitivity	Specificity	AUC
**Clinical**	10fold_CV*	0.660	0.700	0.687	0.751
	Open test*	0.700	0.712	0.692	0.768
**CBC**	10fold_CV**	0.660	0.700	0.517	0.669
	Open test**	0.610	0.675	0.498	0.623
**BCM**	10fold_CV**	0.470	0.632	0.292	0.505
	Open test**	0.550	0.656	0.293	0.535
**Clinical** + **CBC**	10fold_CV*	0.680	0.730	0.705	0.769
	Open test*	0.700	0.739	0.709	0.775
**Clinical** + **CBC** + **BCM**	10fold_CV	0.700	0.739	0.719	0.783
	Open test	0.710	0.742	0.711	0.786

Comparisons of AUCs between the combined features and other feature groups were performed using T-tests based on 1000 permutations. A statistically significant difference is noted: *P-value < 0.05; **P-value < 0.001. *CBC* Complete blood count, *BCM* Blood biochemical marker.

Classification performance for the entire dataset was displayed in Table 3. Clinical features produced good AUC and sensitivity, while CBC biomarkers only obtained modest AUC. Besides, BCMs yielded the lowest performance. Combining all three domains of features got the optimal AUC (10-fold CV: 0.850, Open test: 0.889) and sensitivity (0.816, 0.850). In particular, combined features achieved a boost in the specificity performance (0.831, 0.816), which is vital in clinical practice settings. The levels of both AUCs of the clinical features and the combined features could be considered as good.

Classification performance for samples of disease duration ≤3 years is displayed in Table 4. The overall performance followed the same pattern of performance on the entire samples (Table 3). Clinical features outperformed among the three types of features. Interestingly, the combined features achieved a higher improvement of AUC over the clinical features (10-fold CV: 0.795 vs. 0.829; Open test: 0.817 vs. 0.859), in comparison with AUC improvement on the entire samples (0.830 vs. 0.85; 0.875 vs. 0.889). Besides, accuracies on this sub-dataset were relatively higher and did not subject much to performance changes as the other metric criteria, potentially due to the heavily imbalanced labels (UPD: 681, BPD:50). Similarly, both AUCs of the clinical features and the combined features can be considered as in the level of good.

Table 5 shows the classification performance for samples with disease duration >3 years. The sharp drop in performance on this subset is probably due to the reduced sample size and a long tail of disease duration (ranging from >3 years to >10 years). The optimal AUC was 0.786 produced by the combined features. The AUCs of the clinical features and the combined features could be considered as fair, which may still be helpful to diagnosis in a practical setting.

#### Feature contribution

As illustrated in Tables 3–5, when comparing the AUC of using the entire feature set with other feature sets, statistically significant differences can be observed, demonstrating that each independent feature set (i.e., clinical features, CBC, and BCM) makes statistically significant contribution.

#### Examination of influence from other clinical covariates

Furthermore, to examine the influence of covariant clinical features on differential diagnostic performance, we further added age and gender into the feature set. The performance of the resulting differential diagnosis models was compared with the original feature set. Adding age as feature did not affect performance; this clinical variable could actually be calculated directly by adding age of onset and disease duration. On the other hand, adding gender as a feature significantly decreased performance, with an AUC of 0.862 (vs. 0.889) for the entire cohort, 0.843 (vs. 0.859) for samples with disease duration ≤3 years, and 0.755 (vs. 0.786) for samples with disease duration >3 years. Performance comparisons confirmed that the two covariant features (age and gender), which may have influence on blood biomarker levels, did not contribute positively to the differential diagnosis, and that the current feature set combining clinical variables (age of onset and disease duration) and blood biomarkers has a strong differential diagnosis ability.

## Discussion

The clinical manifestations of UPD and BPD are similar, especially during the depressive episodes of BPD^53^. A comprehensive analysis has been given to medical history, course characteristics, clinical symptoms, and physical, mental, and laboratory examinations, in terms of their statistical differences between UPD and BPD^54^. Psychiatric symptoms and mental disorder rating scales have issues of subjectivity and inconsistency. On the other hand, biomarkers from genetic/omics-related testing and neuroimaging examinations are expensive and have low patient coordination/coverage in real-world settings^55,56^. Therefore, previous studies using features from other domains were conducted on relatively small sample sets, prone to over-fitting and lack of validation in large-scale populations with synthetic heterogeneity^14,33–35,57,58^. Notably, although one or more of the above characteristics can help distinguish UPD from BPD, the current identification performance is not sufficient for practical use^59^. Therefore, it is necessary to find objective and easily accessible features to establish a high-performance differential diagnosis model with wide patient coverage and wide dissemination. Besides, combining differential traits/biomarkers from multiple domains is encouraged to better represent population heterogeneity originating from different aspects^48,49^.

To the best of our knowledge, this discriminative study of UPD and BPD is the first to combine blood-biological data and data of illness courses, with the largest sample set of 1,160 participants. We developed an integrated framework of machine learning to discriminate patients with UPD from BPD. The main findings of this study are described below: (1) using a combination of blood biological features and clinical features of disease course for the classification, the best performance was achieved, with an AUC of 0.889, a sensitivity of 0.831, a specificity of 0.839 and an accuracy of 0.863. (2) the most discriminative features include selected CBC biomarkers (WBC, PLR, MONO, LYMPH, NEUT Ratio, MCHC, BASO Ratio, LYMPH Ratio), BCMs (albumin, calcium, potassium, chlorine, HCT, LDL, HDL) and clinical features (disease duration, age of onset).

The contributions of this study are two folded: (1) Computationally, advanced machine-learning techniques can take full advantage of large, high-dimensional datasets with multi-domain features, comprehensive representation of population heterogeneity and good differential diagnostic performance. In particular, the best algorithm employed in this study, XGBoost, provides state-of-the-art performance for numerical and categorial features by capturing efficient interactions between them. (2) Clinically, this study validates the importance of clinical features of age of onset and disease duration, as well as significant CBC biomarkers and BCMs as objective and quantitative features for accurate differential diagnosis between UPD and BPD. The differential contributions of these features/biomarkers are supported by real-world sample distributions of UPD and BPD participants (Fig. 4), importance analysis using feature extraction algorithms (Fig. 5), tests of between-subjects effects (Table 2), and statistical comparisons of differential diagnosis performance in this study (Tables 3–5). These features are particularly important in patients with a short course of disease who have no overt symptoms or only observed depressive symptoms (as most BD patients exhibit depressive symptoms during their early episodes)^10,59,60^. Automated diagnostic tools will greatly facilitate early BD detection, thereby preventing or delaying disease onset and improving clinical outcomes^10,59,60^. Practically, these features are readily available in routine clinics covering a wide range of patients. This is critical to promote automated diagnostic tools by leveraging large-scale, low-cost data resources in diverse clinical settings. Looking into the importance of specific blood biomarkers in samples of disease durations ≤3 years and >3 years, WBC and MONO remained informative across different disease durations. Meanwhile, NEUT, BASO Ratio, HCT and LYMPH, and albumin were more indicative in the short course (≤3 years), whereas NLR and chlorine were more indicative in the longer course (>3 years). Visualizing the output tree of XGBoost using SHAP can provide interpretable, personalized risk factors for each specific patient diagnosis^61^.

Interestingly, the effective rate (AUC) of the model could reach 0.875 when the clinical features of disease course were used alone, and after adding blood-related indicators, the effective rate increased to 0.889. Notably, previous studies usually constructed cohorts with equal samples of UPD and BPD, which may not reflect their incidence in practical settings. This study measured and reported the differential performance on the original proportion of patients (UPD: 918, BPD: 242). In particular, this study also examined the performance of samples with different disease durations and different prevalence rates (i.e., ≤3 years, UPD: 681, BPD: 50 and >3 years, UPD: 237, BPD: 192) for the first time (Tables 4–5), to further understand the effectiveness of the differential diagnosis model on patients at early/later stages of the disease course. As illustrated in Tables 3–5, each independent feature set (i.e., clinical features, CBC biomarkers, and BCMs) made a statistically significant contribution to the optimal performance. In particular, BCM made a significant contribution when added to the feature set (*P* < 0.001 for the entire cohort, *P* < 0.001 for disease duration ≤3 years, *P* < 0.05 for disease duration >3 years), despite a poor performance on its own. Moreover, the significant contributions of clinical features and blood biomarkers on differential diagnosis were consistent across different disease durations (Tables 3–5), demonstrating their differential ability in (sub-)populations of different heterogeneity and prevalence rates.

Another interesting finding is that the top-ranked blood-related indicators were mainly from BCMs, including albumin, LDL, and potassium, instead of CBC biomarkers. To interpret the potential reasons of why these biomarkers are salient features, the association between medical conditions related to these biomarkers and potential influence to mental states is discussed here: (1) As mentioned earlier, patients with mood disorders have decreased antioxidant capacity and oxidative stress damage^62^. The detection of non-enzymatic antioxidants such as albumin can be conducted conveniently and can be used to monitor the antioxidant level of the body. Previous studies have found that plasma albumin concentrations were lower in the UPD group than in the mania group^44^. The results of this study provide additional evidence that albumin was lower in the UPD group (8.701 ± 3.370) compared to the BPD group (8.808 ± 2.677). (2) Furthermore, while this study found higher LDL levels in BPD (153.781 ± 28.961) compared to UPD (148.952 ± 28.897), other studies have reported inconsistent results^46^. Abnormal LDL levels have been reported to be associated with multiple medical commodities in UPD and bipolar disorder, such as metabolic syndrome and vascular disease^63,64^. High LDL levels are prone to atherosclerosis and accelerate the development of cardiovascular and cerebrovascular sclerosis^64,65^. (3) As for potassium, previous studies have reported lower plasma potassium levels in UPD patients compared with controls^66^. No statistical comparisons with BPD patients have been reported yet. This study found that the plasma potassium levels were higher in UPD patients (6.734 ± 3.160) than in BPD patients (6.343 ± 3.946). Low plasma potassium levels are associated with higher risk of mood swings^67^, potentially through mechanisms affecting intracranial ion channels^68^.

### Limitations and future work

(1) One limitation is that some blood biochemical markers were removed from the original feature set due to more than 30% missing values, which may have the potential to be important features and further improve the performance. (2) In addition to performances of different disease durations as analyzed here, it would worth looking into the performance and feature contributions based on other sample stratifications such as socio-demographic characteristics, severity levels of disorders and psychotropic medication usages in the near future, to examine the model stability and feature importance from different aspects. (3) In our pilot study, we have examined other clinical features reported in the literature to be associated with specific types of depression^9,10,18,59,69^, such as family history of bipolar disorder, number of previous depressive episodes, and other characteristics of depressive episodes. However, none of them could obtain performance comparable to course features (age of onset and disease duration), or contribute positively to the current feature set. Data of only one site was used for experiments in this study, although the sample size and distributions were representative as discussed above, the model and other potentially useful features need to be examined on more samples from different settings in the next step. (4) In addition to performance of different disease durations as analyzed here, it would worth looking into the performance and feature contributions based on other sample divisions such as socio-demographic characteristics and age of onset in the near future, to examine the model stability and feature changes from different aspects. (5) One important direction in the next step is to further study the essential mechanisms behind the ability of blood biomarkers to distinguish UPD and BPD. (6) It would be of significant clinical importance to examine indicated blood markers from this study in a control group of UPD and BPD patients. The recruit work is under plan and a further validation/analysis will be carried out in the next step. (7) As found in the feature importance analysis, distinct blood biomarkers may be more indicative of different disease durations. This may suggest that, given the overall stability of the model, changes in biomarkers across disease duration and age groups should be investigated. (8) Another future direction is to use patient longitudinal data to cluster potential depression subtypes with different progression patterns and to build personalized models of biomarker changes over the course of the disease for better patient stratification, more precise diagnosis and tailored interventions.

In summary, there is a lack of objective features for the differential diagnosis of unipolar and bipolar depression. We investigated whether a combination of hematologic biomarkers and clinical features could accurately classify unipolar and bipolar depression using machine learning methods. Experimental results demonstrated that hematologic biomarkers and onset features are reliable information that could be easily accessible in clinical settings to improve diagnostic accuracy.

### Supplementary information

Supplementary information

## Data Availability

The data used for experiments and analysis in this study are available from the corresponding author upon reasonable request.

## Code availability

Codes of machine learning algorithms used in this study are publicly available at https://github.com/scikit-learn/scikit-learn. The code used for SHAP analysis is available at https://github.com/slundberg/shap.
