# Supplementary material for: Optimizing multi-domain hematologic biomarkers and clinical features for the differential diagnosis of unipolar depression and bipolar depression
Source: Npj Ment Health Res. 2023 Apr 10;2:4. doi: 10.1038/s44184-023-00024-z (PMC10955811; doi:10.1038/s44184-023-00024-z)
Supplement: Supplementary file 1 — Supplementary information [file 44184_2023_24_MOESM1_ESM.docx]

**Supplement**

**Table 1**. Abbreviations of blood biomarkers used in this study and their full names.

| Abbreviation | Full name |
| --- | --- |
| Biomarkers of the erythrocyte system | |
| RBC | red blood cell count |
| HCT | hematocrit |
| MCV | mean corpusular volume |
| PDW | red blood cell volume distribution width |
| RDW-CV | red blood cell volume distribution width - coefficient of variation |
| RDW-SD | red blood cell volume distribution width - standard deviation |
| HGB | hemoglobin |
| MCH | mean corpusular hemoglobin |
| MCHC | mean corpusular hemoglobin concerntration |
|  |  |
| Biomarkers of the leukocyte system | |
| WBC | white blood cell count |
| MONO | monocyte count |
| MONO ratio | monocyte percentage |
| NEUT | Neutrophil count |
| NEUT ratio | Neutrophil percentage |
| BASO | basophil count |
| BASO ratio | basophil percentage |
| EO | eosinophil count |
| EO ratio | eosinophil percentage |
| LYMPH | lymphocyte count |
| LYMPH ratio | lymphocyte percentage |
|  |  |
| Biomarkers of the platelet system | |
| PLT | platelet count |
| MPV | mean platelets volume |
| P-LCR | platelet larger cell ratio |
|  |  |
| Blood count-related inflammatory markers | |
| NLR | neutrophil-to-lymphocyte ratio |
| PLR | platelet-to-lymphocyte ratio |
| MLR | monocyteto-lymphocyte ratio |
|  |  |
| Markers of blood lipids | |
| HDL | high density lipoprotein |
| LDL | low density lipoprotein |

**Figure 1**. Feature importance calculated as mean SHAP values averaged from running ten-fold cross validation for 1,000 times and normalized to the range of [0,1].

1. ROC curves for the entire sample set.

1. ROC curves for samples of disease duration ≤ 3 years.

1. ROC curves for samples of disease duration > 3 years.

**Figure 2**. **ROC curves of using clinical features, hematologic biomarker and combined features on the open test data, respectively**: (a) ROC curves for the entire sample set. (b) ROC curves for samples of disease duration ≤ 3 years. (c) ROC curves for samples of disease duration > 3 years.

**Table 2.** Classification performances of logistic regression using clinical features, hematologic biomarkers and their combination, based on the entire cohort.

| **Feature** | **Dataset** | **Accuracy** | **Sensitivity** | **Specificity** | **AUC** |
| --- | --- | --- | --- | --- | --- |
| **Clinical** | 10fold_CV | 0.800 | 0.800 | 0.763 | 0.808 |
|  | Open test | 0.809 | 0.812 | 0.779 | 0.856 |
| **Common blood count** | 10fold_CV | 0.663 | 0.667 | 0.676 | 0.675 |
|  | Open test | 0.639 | 0.622 | 0.629 | 0.644 |
| **Blood biochemical marker** | 10fold_CV | 0.531 | 0.515 | 0.496 | 0.500 |
|  | Open test | 0.573 | 0.567 | 0.503 | 0.523 |
| **Combined** | 10fold_CV | 0.822 | 0.806 | 0.811 | 0.848 |
|  | Open test | 0.839 | 0. 851 | 0.813 | 0.870 |

**Table 3.** Classification performances of SVM using clinical features, hematologic biomarkers and their combination, based on the entire cohort.

| **Feature** | **Dataset** | **Accuracy** | **Sensitivity** | **Specificity** | **AUC** |
| --- | --- | --- | --- | --- | --- |
| **Clinical** | 10fold_CV | 0.810 | 0.802 | 0.769 | 0.817 |
|  | Open test | 0.816 | 0.815 | 0.786 | 0.865 |
| **Common blood count** | 10fold_CV | 0.668 | 0.669 | 0.680 | 0.685 |
|  | Open test | 0.647 | 0.625 | 0.638 | 0.666 |
| **Blood biochemical marker** | 10fold_CV | 0.534 | 0.515 | 0.500 | 0.503 |
|  | Open test | 0.578 | 0.567 | 0.509 | 0.525 |
| **Combined** | 10fold_CV | 0.826 | 0.809 | 0.815 | 0.857 |
|  | Open test | 0.852 | 0. 861 | 0.816 | 0.879 |

**Table 4.** Classification performances of random forest using clinical features, hematologic biomarkers and their combination, based on the entire cohort.

| **Feature** | **Dataset** | **Accuracy** | **Sensitivity** | **Specificity** | **AUC** |
| --- | --- | --- | --- | --- | --- |
| **Clinical** | 10fold_CV | 0.805 | 0.800 | 0.765 | 0.810 |
|  | Open test | 0.810 | 0.814 | 0.780 | 0.858 |
| **Common blood count** | 10fold_CV | 0.664 | 0.667 | 0.678 | 0.68 |
|  | Open test | 0.641 | 0.623 | 0.631 | 0.645 |
| **Blood biochemical marker** | 10fold_CV | 0.532 | 0.515 | 0.495 | 0.499 |
|  | Open test | 0.575 | 0.566 | 0.506 | 0.521 |
| **Combined** | 10fold_CV | 0.822 | 0.804 | 0.813 | 0.85 |
|  | Open test | 0.842 | 0. 850 | 0.816 | 0.870 |

**Table 5**. Comparison of each blood biomarker between the two groups of unipolar and bipolar depression. Two sets of p-values are reported: p is obtained from T-tests; p* is obtained from Tests of between-subjects effects with each blood biomarker as dependent variable and clinical variables as covariates. Statistically significant differences are observed for each blood biomarker between the two groups, regardless of whether clinical variables are considered as covariates. This demonstrates the independent ability of each biomarker to differentiate between unipolar and bipolar depression.

| **Biomarker** | **Unipolar depression** | **Bipolar depression** | **p** | **p*** |
| --- | --- | --- | --- | --- |
| **WBC** | 5.87 ± 1.59 | 6.67 ± 2.01 | .000 | .000 |
| **PLR** | 121.99 ± 46.76 | 111.24 ± 43.52 | .001 | .002 |
| **MONO** | .46 ± .15 | .52 ± .18 | .000 | .000 |
| **Albumin** | 40.77 ± 3.47 | 41.94 ± 4.11 | .000 | .000 |
| **LDL** | 2.47 ± .79 | 2.69 ± .88 | .000 | .000 |
| **Potassium** | 4.00 ± .33 | 4.07 ± .31 | .001 | .000 |
